# Current overview on the genetic basis of key genes involved in soybean domestication

**DOI:** 10.1007/s42994-022-00074-5

**Published:** 2022-07-02

**Authors:** Sijia Lu, Chao Fang, Jun Abe, Fanjiang Kong, Baohui Liu

**Affiliations:** 1 Innovative Center of Molecular Genetics and Evolution, School of Life Sciences, Guangzhou University, Guangzhou, 510006 China; 2 Guangzhou Key Laboratory of Crop Gene Editing, Guangzhou University, Guangzhou, 510006 China; 3 Research Faculty of Agriculture, Hokkaido University, Sapporo, Hokkaido 060-0808 Japan

**Keywords:** Soybean, Domestication, Diversity, Gene

## Abstract

Modern crops were created through the domestication and genetic introgression of wild relatives and adaptive differentiation in new environments. Identifying the domestication-related genes and unveiling their molecular diversity provide clues for understanding how the domesticated variants were selected by ancient people, elucidating how and where these crops were domesticated. Molecular genetics and genomics have explored some domestication-related genes in soybean (*Glycine max*). Here, we summarize recent studies about the quantitative trait locus (QTL) and genes involved in the domestication traits, introduce the functions of these genes, clarify which alleles of domesticated genes were selected during domestication. A deeper understanding of soybean domestication could help to break the bottleneck of modern breeding by highlighting unused genetic diversity not selected in the original domestication process, as well as highlighting promising new avenues for the identification and research of important agronomic traits among different crop species.

## Introduction

Soybean (*Glycine max* (L.) Merr.) is an economically important leguminous crop that provides more than 25% of the protein used for food and animal feed worldwide (Graham and Vance 2003). Cultivated soybean was domesticated from wild *Glycine soja* (Siebold. & Zucc.) in East Asia around 6000–9000 years ago (Carter et al. 2004; Kim et al. 2011). The process of domestication is long, and comes with the conscious and unconscious artificial selections that result in drastic morphological and physiological changes between the domesticated plants and their wild progenitors, known as the “domestication syndrome” (Hammer 1984). For soybean, the typical traits of the domestication syndrome include the loss of seed dormancy and dispersal ability, a change of plant architecture from twining more branched growth form to upright fewer-branched form, and a change in seed morphology from small seeds covered by a powdery bloom to larger and colorful seeds (Liu et al. 2007). After the initial domestication, *G. gracili* (semi-wild soybeans) and landrace experienced the diversification to spread and improve adaptability to different agro-ecological and cultural environments. During this domestication and diversification, many of the traits changed, but the molecular mechanisms underlying these changes are not yet be fully elucidated. The genes that function in domestication traits are positively selected, and at least one causative mutation was fixed within the crop population during domestication to be defined as a domesticated gene (Meyer and Purugganan 2013). In the following section, we will summarize the molecular bases of genes that were involved in the process of domestication in soybean.

## Shattering

Wild soybeans colonize new areas by dispersing their seeds long distances through seed shattering, which reduces the competition between parents and offspring to increase their chances of survival. In an agricultural context however, seed shattering results in the loss of seeds and yield, and is therefore unfavorable. The reduction of seed dispersal, especially the loss of seed shattering, is a hallmark of domestication in soybean. Many genes involved in the seed-shattering process have been identified in the model plant *Arabidopsis thaliana*, including *SHATTERPROOF1* (*SHP1*) and *SHP2* (MADS-box genes), *INDEHISCENT* (a basic helix-loop-helix (bHLH) gene), *ALCATRAZ* (a Myc/bHLH gene), and *NAC SECONDARY WALL THICKENING PROMOTING FACTOR1* (*NST1*) and *NST2* (Liljegren et al. 2000; Rajani and Sundaresan 2001; Mitsuda et al. 2005, Sorefan et al. 2009). Some seed-shattering genes have also been identified in crops, such as a trihelix transcription factor–encoding gene in rice (*Oryza sativa*) (Zhou et al. 2012), a gene encoding an APETALA2 -type transcription factor in wheat (*Triticum aestivum*) (Simons et al. 2006), and a YABBY transcription factor–encoding gene in sorghum (*Sorghum bicolor*) (Lin et al. 2012).

The first pod-shattering quantitative trait locus (QTL) to be identified in soybean, which could account for 44% of the variation in this trait, was identified on chromosome 16 using a recombinant inbred line (RIL) population derived from a cross between Young (pod-shattering resistance) and PI 416937 (pod-shattering sensitive) (Bailey 1997). Around this interval, Funatsuki et al. (2006) detected one major QTL controlling pod shattering between the simple sequence repeat (SSR) markers Sat_093 and Sat_366 within the RIL population derived from the cross between Hayahikari (high shattering resistance) and Toyomusume (low shattering resistance). Funatsuki et al. (2008) next used four different populations in different environments to further demonstrate that the interval between two markers (Sat_093 and Sat_366) contains one major gene controlling shattering resistance, and named this QTL *qPDH1*. *qPDH1* has been detected in different populations in many studies, providing evidence that it is a stable QTL that plays an important role in soybean (Funatsuki et al. 2011). In studies of the one line found to be heterozygous at *qPDH1*, which was derived from the RIL population from the research in 2005 (Funatsuki et al. 2005), the interval of *qPDH1* was limited to a 134-kb region (Funatsuki et al. 2014). Among this interval, no gene showed significant sequence homology with the *Arabidopsis* pod-shattering genes that were previously identified (Suzuki et al. 2010). In *Arabidopsis*, most of the shattering-resistance genes increase the binding strength of the seed pod abscission layers, which form at the binding sites of the pod walls. These layers accumulate the dehiscing force during the maturation process as the pods dry. When the dehiscing force exceeds the binding strength of the pod walls, pod shattering occurs (Ferrandiz et al. 2000; Liljegren et al. 2000; Rajani and Sundaresan 2001; Ogawa et al. 2009). In addition to the lack of a *qPDH1* homolog in *Arabidopsis*, the differences between the fruit structures of *Arabidopsis* and soybean may further indicate that *qPDH1* contains a new gene or genetic mechanism that controls pod shattering, but one which is unlikely to influence the pod wall binding strength (Christiansen et al. 2002; Lenser and Theißen 2013; Tiwari and Bhatia 1995).

Using a combination of the linkage and association mapping, Gao et al. (2013) further delimit the *qPDH1* locus to a 47-kb region containing only two predicted genes, *Glyma16g25600* (*Glyma.16G141500*, the transcript name in the genome of Willimas 82 (Wm82.a2.v1), the same below) and *Gm16g25610* (*Glyma.16G141600*). *Glyma.16G141500* encodes a bZIP-type transcription factor and was originally believed to be the most likely *qPDH1* candidate gene because most of the known domestication genes are involved in transcriptional regulation (Doebley et al. 2006). Then the candidate region was further narrowed to 20 kb (Funatsuki et al. 2014). Using a combined sequencing and expression analysis, *Glyma16g25580* (no corresponding gene in the soybean genome for Wm82.a2.v1), which encodes a dirigent (DIR)-like protein, was identified as the candidate gene of *qPDH1*. The single-nucleotide polymorphism (SNPs) occurring in this gene is an A in shattering-susceptible cultivars and a T in shattering-resistant cultivars, leading to the premature termination of the protein. This gene was therefore named *Pdh1*. DIR proteins are known to mediate stereoselective coupling, disease resistance, and the formation of lignin-based Casparian strips in roots (Liu et al. 2008b; Pickel et al. 2010; Kim et al. 2012; Hosmani et al. 2013; Funatsuki et al. 2014). Funatsuki et al. (2014) demonstrated that the overexpression of *Pdh1* during the initiation of lignin deposition in the inner sclerenchyma tissue, the site of thick secondary cell wall formation, promotes pod dehiscence, a novel function for the DIR superfamily; however, this candidate gene, *Glyma16g25580*, was not present in the new version of the soybean genome assembly.

By performing a morphological observation of the mature fruit, Dong et al. (2014) revealed that the vascular bundle valves at the ventral suture were different between the pods of the cultivar HEINONG 44 and wild soybean ZYD00755. An anatomical examination showed that HEINONG 44 has thicker fiber cap cells in the secondary wall than ZYD00755. Based on the phenotype comparison and homolog analyses, 13 candidate genes were identified, orthologs of which function in the regulation of seed shattering in *Arabidopsis*. Among those genes, *Glyma04g39210* (*Glyma.04G214100*) and *Glyma16g02200* (*Glyma.16G019400*) exhibited a dramatic reduction in the nucleotide polymorphism between wild accessions and landraces, suggesting that these two genes mainly participate in the regulation of soybean pod shattering. *Glyma.16G019400*, which is located in a known QTL region associated with pod dehiscence was named as *SHATTERING1-5* (*SHAT1-5*). *SHAT1-5* encodes a NAM, ATAF1/2 and CUC2 (NAC) domain transcription factor homologous to *NST1/2* in *Arabid*opsis, which was reported to activate secondary cell wall thickening. A 20-bp deletion in the promoter (− 4.0 kb) of *SHAT1-5* in HEINONG 44 resulted in its higher expression in this cultivar than in ZYD00755, which promoted the formation of a thicker secondary cell wall in the cultivated soybean and resulted in shattering resistance. To further demonstrate that *SHAT1-5* controls pod shattering*,* the expression level of *SHAT1-5* in the F_2_ population derived from a cross between HEINONG 44 and ZYD00755 was also tested. *GmSHAT1-5* (the allele from the HEINONG 44) co-segregated with the heavily thickened fiber cap cells, and was expressed to a higher expression level in *GmSHAT1-5* homozygotes than the *GsSHAT1-5* allele (from ZYD00755) in its corresponding homozygote. These finding all suggest that at the molecular level all point to the result that *GmSHAT1-5* effectively controls the shattering-resistant trait. To provide further evidence for this, soybean genomics were also studied. The noncoding nucleotide diversity of this gene was severely reduced from the wild accessions to the landraces; the landraces were all combined into a single clade in a phylogenetic analysis. *SHAT1-5* was located in a ~ 116-kb selective sweep in these lines, indicating that it had undergone artificial selection and was a domesticated gene.

Using high-throughput SNP genotyping systems, Lee et al. (2017) identified two SNPs in *Glyma.16g141600* that resulted in amino acid substitutions, and which clearly discriminated pod shattering-resistant varieties from pod shattering-susceptible varieties among 38 of Korean soybean cultivars.

In total, four different genes located in close proximity on the soybean chromosome 16 were reported to control pod shattering, but only two of them were further researched on the molecular mechanism and met the standard of “domesticated genes” and were listed in Table 1. In addition, some markers were also reported to be helpful for identify the shatter resistant accessions on the chromosome 16 (Miranda et al. 2019). The interaction among these genes may cover the effect of the respective genes (Liljegren et al. 2000; Nishizawa et al. 2006). So, these genes may be true and coordinatively regulate pod shattering. In the domestication of soybean, the cultivars would have been selected for different environmental conditions, which can also have a strong influence on pod shattering. First, several shattering-resistance genes were selected, such as *SHAT1-5*, to achieve shattering resistance in its native humid climate. When soybean was transported from this humid climate to drier regions, other genes, such as *Pdh1*, were subsequently fixed (Funatsuki et al. 2014).

**Table 1:** Genes of published in soybean domestication

Trait	Name	Locus	Conserved domain or function	Mutant type	References
Shattering	*qPDH1*	*Glyma16g25580*	Dirigent (DIR)-like protein	SNP in CDS	Funatsuki et al. 2014
	*SHAT1-5*	*Glyma.16G019400*	NAC (NAM, ATAF1/2 and CUC2) domain transcription factor	Indel in promoter	Dong et al. 2014
Dormancy	G	*Glyma.01g198500*	CAAX amino-terminal protease protein	SNP in CDS	Wang et al. 2018
Hard seeds	*GmGH9B8*	*Glyma.02G269400*	Endo-1,4-β-glucanase	SNP in CDS	Jang et al. 2015
	*GmHs1-1*	*Glyma.02G269500*	PhoD-like phosphatase	SNP in CDS	Sun et al. 2015
Seed coat shininess	*B1*	*Glyma.13g241700*	Transmembrane transporter like protein	SNP in CDS	Zhang et al. 2018
Seed oil content	*GmSWEET39/ GmSWEET10a*	*Glyma.15g049200*	Sugar efflux transporter for intercellular exchange	Indel and SNP in promoter and SNP in CDS	Wang et al. 2020; Miao et al. 2020
Flowering	*Tof12*	*Glyma.12g073900*	Two-component response regulator-like APRR3	SNP in CDS	Lu et al. 2020

Pod shattering is implemented by the cell–cell separation, rather than rupture of the cells (Spence et al. 1996). In plant species with pod-fruit type, pod shattering was influenced by the balance between the dehiscing force of abscission layers at the valves and binding strength of the pod walls. The soybean accessions used in the above studies were collected from regions with different climates. The environmental stress may influence the development of a dehiscence zone at the valve replum boundary, the lignification of cells to the dehiscence zone, and some other contributing factors that regulate the pod shattering. So different genes were selected. This was one reason for explaining why different genes were detected in the above research. The other reason was that differing methods for detecting phenotypes were used. For example, for the fine mapping of *Glyma.16G141500*, the recombinant genotype was cultivated in a growth chamber rather than the field, and shattering resistance was recorded at a 30% relative humidity rather than a heat treatment (Funatsuki et al. 2014). By contrast, in the study identifying *SHAT1-5*, the researchers used both natural (field) conditions and experimental conditions (37 °C for 4 d) to quantify shattering (Dong et al. 2014). Kang et al. (2005) used an oven to dry the pod at 40 °C for 24 h. Different methods will have resulted in different pod water contents, which has a major impact on pod shattering. Accordingly, more research into the molecular regulation of seed shattering is required.

## Dormancy

An intact viable seed is not able to complete germination until certain conditions are met, which is defined as seed dormancy. This process means the seed case protects the embryonic plant until conditions are optimal for the new plant’s survival. This trait can be a disadvantage for crops however, because dormancy can result in differing emergence times, making harvest more difficult and reducing the final yield. The loss or weakening of seed dormancy is often a fundamental requirement for agriculture, especially for crops harvested mechanically; therefore, dormancy is a typical domestication syndrome trait (Sugimoto et al. 2010; Olsen and Wendel 2013). Based on when it occurs, dormancy can be divided into primary and secondary dormancy (Hilhorst 1995). Primary dormancy mainly exists in the process of seed development and maturation (Kucera et al. 2005), while secondary dormancy can only occur after seed dispersal (Leubner-Metzger 2006). Baskin et al. (2004) proposed a modified version of the system of classification for seed dormancy, dividing it into five classes: physiological, morphological, morphophysiological, physical, and combinational dormancy. Among these five classes, physiological dormancy is the most common (Pallais 1995, Li and Foley 1997, Foley and Fennimore 1998, McKibbin 1999, Koornneef et al. 2002).

Dormancy is a complex phenomenon controlled by a large number of genes, and it is affected by both developmental and environmental factors (Bewley 1997; Koornneef et al. 2002). Many of the genes associated with dormancy were first identified in *Arabidopsis*. Most of them participate in hormonal pathways; however, the mechanism has yet to be fully elucidated (BirgitKucera 2005). In soybean, dormancy involves both physiology and physical dormancy, making it more difficult to identify the candidate genes (Leubner-Metzger 2006).

Using a genome-wide association study, Wang et al. (2018) identified the *G* gene for the green seed coat color (*Glyma.01g198500*), which encodes a CAAX amino-terminal protease protein. The results of *Fst*, nucleotide diversity, cross-population composite likelihood ratio, and haplotype homozygosity analyses of the SNP site showed that the *G* gene is located in a selective sweep region between wild and cultivated soybeans, suggesting that *G* is a domestication-related gene. Wild soybeans produce black coat color to prevent the seeds from the predation, but possess the *G* green seed coat genotype (Porter 2013). This suggests that *G* may have other functions targeting domesticated traits. Wang et al. (2018) next used germination and dormancy-breaking experiments to confirm that *G* functions in dormancy. Further analysis on molecular mechanism of *G* suggested that the G protein may interact with nine-cis-epoxycarotenoid dioxygenase 3 (NCED3) and phytoene synthase (PSY), the key enzymes involved in abscisic acid (ABA) biosynthesis. This interaction may result in the production of more ABA, which induces and maintains dormancy. Finally, they proposed that the parallel selection of the mutant *g* allele during crop domestication, conferring reduced ABA content and weakened seed dormancy, promotes seeds to germinate uniformly and thus is advantageous for crop management.

In *Arabidopsis*, *AtDOG1* play important role in controlling dormancy (Nakabayashi et al. 2015; Cyrek et al. 2016). It interacted with ABA signal pathway genes including *AHG1* (ABA-HYPERSENSITIVE GERMINATION 1) and *AHG3* to regulate dormancy and germination (Née et al. 2017). In order to understand the genetic information of *DOG1* family in soybean, Yang et al (2020) identified 40 members of *DOG1-Like* (DOG1L) family in soybean genome. Among these genes, *GmDOG1-L37* was closest to *AtDOG1*, and had the highest expression in seeds when compared with the other tissues. During the seed developments, the expression of *GmDOG1-L37* continues to increase. All of these suggest that *GmDOG1-L37* is the *GmDOG1* gene in soybean.

## Hard seeds

Besides the physiological dormancy, which was controlled by *G*, two other genes were reported to function in physical dormancy. Most wild soybeans form hard seed coats, which are impermeable to maintain physical dormancy. From the perspective of morphology, minute cracks are situated in the subcuticular layer or on the dorsal side of the seed coat to enable water to enter the seed and break dormancy (Dalling et al. 2011; Paulsen et al. 2013). Other studies found that the contents of xylans, hydroxylated fatty acids, and calcium in the seed coat controlled its level of permeability (Ma et al. 2004; Shao et al. 2007; Paulsen et al. 2013). Previous studies of the hard seed coat focused on identifying the associated QTLs (Keim et al. 1990; Sakamoto et al. 2004; Watanabe et al. 2004; Liu et al. 2007; Zhang et al. 2008a). One stable QTL was detected on chromosome 2 (*qHS1*) in several studies, Jang et al. and Sun et al. further explored this region to identify the candidate genes, respectively (Keim et al. 1990; Watanabe et al. 2004; Liu et al. 2007; Zhang et al. 2008a; Jang et al. 2015; Sun et al. 2015).

Jang et al. (2015) backcrossed a hard-seed wild soybean with a recurrent parent, Tachinagaha (TA), which produces permeable seeds, to obtain the near-isogenic line (NIL) TA-HS, containing the hard-seed allele in the TA background. They achieved this using the marker Satt459, which was reported to be the closely linked marker for *qHS1*. The surface of the palisade layer of the TA seed coat contained many cracks, favorable for water infiltration, while the hard seed coat of TA-HS had many pits rather than cracks on its surface. Further observation found that the cracks in TA showed a ladder like structure, in which the palisade cells are partly connected, promoting water access and absorption; by contrast, the pits of the TA-HS seed coats were closed. This result was consistent with a previous finding that permeable and impermeable seeds always formed cracks and pits on the seed coat, respectively (Ma et al. 2004).

Combining the phenotypes and genotypes of the recombinants, Jang et al. (2015) fine mapped the candidate interval to a 93-kb region. The sequence and expression analyses showed that a SNP in *Glyma02g43680* (*Glyma.02G269400*) occurred in the substrate-binding cleft region, causing an isoleucine residue in TA to be replaced by a serine residue in TA-HS. This gene encodes an endo-1,4-β-glucanase gene, which hydrolyzes β-1,4-glucosyl linkages (Henrissat 1991; Molhoj et al. 2002; Libertini et al. 2004). The transformation of the TA-HS allele into a cultivar with a permeable seed coat promoted the accumulation of β-1,4-glucan in the outer layer of palisade cells and reduced seed coat permeability. The observed accumulation of β-1,4-glucan in the seed coats of the NILs confirmed that the serine in TA-HS is responsible for its increased accumulation of β-1,4-glucan in the outer seed coat layers. *Glyma.02G269400* was therefore considered the candidate gene for *qHS1* and named *GmGH9B8,* according to the standardized nomenclature (Urbanowicz et al. 2007). In addition, the association analysis between the SNP in *GmGH9B8* and the seed coat permeability trait suggested that this SNP may be a useful marker of seed permeability.

Sun et al. (2015) also fine mapped the *qHS1* region, identifying a candidate interval of 22 kb. Only two genes were annotated in this interval, according to the Williams 82 reference genome. A sequencing analysis among *G. soja* accessions and a soybean cultivar (Williams 82) indicated that only a C-to-T point mutation in *Glyma02g43700* (*Glyma.02G269500*), which resulted in an amino acid change from threonine to methionine, could explain the difference in seed permeability between Williams 82 and the various *G. soja* accessions. In addition, *Glyma.02G269500* was predicted to encode a calcineurin-like metallophosphoesterase transmembrane protein, was found to be expressed in developing seed coats, and its expression level was much higher in *G. soja* than in Williams 82. *Glyma.02G269500* was therefore considered a candidate gene for *qHS1* and named *GmHs1-1*.

The candidate interval for *GmGH9B8* contained *GmHs1-1*, but *GmHs1-1* was not found to be expressed in the seed coat by Jang et al. (2015). This may because *GmHs1-1* and *GmGH9B8* function in different pathways or at different developmental stages to regulate the hardness of the seed coat. In an analysis of a representative soybean population, Jang et al. (2015) found that 83 of 86 cultivated accessions carried the *Gmhs1-1* (permeable seed) allele, while all six landraces carried *GmHs1-1* (hard-seed) allele. Although the landraces all carried the hard-seed genotype, a lot of cracking was detected on their seed coats, which may be control by *GmGH9B8.* This suggested that *GmGH9B8* may have been selected first because the phenotype was obvious, after which *GmHs1-1* was then selected.

## Seed coat shininess

Most wild soybeans have a powdery bloom on their seed coat, making the seeds less visible to potential predators when they fall to the ground (Wang et al. 2008). The seed coat bloom is derived from the endocarp, where HYDROPHOBIC PROTEIN FROM SOYBEAN (HPS) is biosynthesized, and then deposited on the seed surface (Newell and Hymowitz 1978). HPS is a potentially hazardous allergen, which may help to deter predators, but it can also cause asthma in humans. For this reason, the soybean bloom was eliminated, resulting in variable seed coat lusters in the landraces. Following domestication, most cultivars therefore have shiny seed coats. Previous research found that three loci *B1* to *B3* control bloom development, with *B1* being the most important locus (Chen and Shoemaker 1998; Gijzen et al. 1999; Wang et al. 2016b).

The phenotypic analysis of F_1_ and F_1:2_ seeds derived from crosses between two wild soybeans (bloom) and the cultivar Williams 82 (no bloom) showed that the bloom is mainly controlled by a single gene, which is dominant or partially dominant over the no-bloom phenotype (Zhang et al. 2018). The candidate interval for this bloom gene was reduced to a 14.5-kb region on chromosome 13, which overlaps with the region contained *B1* (Chen and Shoemaker 1998). Two SNPs resulting in amino acid changes were detected among the three parental lines, but only the substitution C to T in the CDS of *Glyma.13g241700* was correlated with the bloom phenotypes; wild soybeans had a C, while Williams 82 had a T. In addition, only this point mutation changed the helix structure of the predicted transmembrane transporter-like protein.

Using a genome-wide association study (GWAS) with 302 resequenced accessions, a QTL associated with seed oil was detected 31 kb downstream of the *B1*(Zhou et al. 2015). This raises the question whether *B1* has a pleiotropic effect on seed oil content. To test this hypothesis, Zhang et al. (2018) selected a population of 70 bloom and 52 no-bloom accessions for GWAS on seed oil content and found one seed oil QTL in the *B1* selective sweep region. These results suggested that *B1* has a pleiotropic effect on seed oil content. Transgenic experiments demonstrated that high expression levels of the *B1* locus in the pod and its endocarp causes the seed coat bloom and reduces the oil content. By contrast, in domestication, the *b1* allele in soybean landraces and cultivars increased the seed oil content and reduced the seed coat bloom. This shows that *B1* not only takes part in the regulation of the bloom, but also controls the seed oil content.

## Seed oil content

Compared with wild soybeans, cultivars have larger seeds with a higher oil content and lower levels of protein (Clemente and Cahoon 2009; Wang et al. 2019, 2020). These traits are influenced throughout the three phases of seed developments: seed set, growth and maturation (Ruan et al. 2012). Seed set is the early stage, laying a foundation for the later stages to determine the seed number, size and likely impacting the yield (Tischner et al. 2003; Weber et al. 2005; Wang and Ruan 2012). The transition of sugars from the liquid endosperm supplies nutrients to the embryo and ensures the seed can reach full maturity (Olsen 2001; Sun et al. 2010). Sucrose is the major form of photosynthetic product, which was transfer to the maternal seed coat via the phloem and then secreted from the seed coat to feed the embryo (Chen et al. 2015). With the help of sugar transporter, especially the membrane-bound sugar transporters, sucrose could fulfill their destiny (Patrick and Offler 2001). The *SWEET* (*Sugars Will Eventually be Exported Transporter*) family members have seven transmembrane domains and function in sugar efflux or influx, playing key roles in phloem loading for long-distance sucrose translocation, pollen nutrition, nectar secretion, seed filling, and many other pathway (Chen et al. 2012, 2015; Sun et al. 2013; Xuan et al. 2013; Yuan and Wang 2013; Lin et al. 2014). The *Arabidopsis* genome contains 17 *SWEET* homologs divided into four clades. Clade III contained the most members (from *AtSWEET 9* to *AtSWEET 15*), all of which are likely to be involved in the cellular efflux of sucrose (Wang et al. 2019). The triple mutant *sweet11;12;15* had severe defects and showed a “wrinkled” seed phenotype and a reduced seed weight (Chen et al. 2015). Another member of Clade III, *SWEET9,* is a nectary-specific sugar transporter, which functions as an efflux transporter to play a key function in nectar production (Lin et al. 2014).

Compared with *Arabidopsis*, soybean has a much larger seed that required more sugars; thus, small changes in their sugar content will have a great influence on the seed development (Wang et al. 2020). In the soybean genome, there are at least 37 *SWEET* members (Wang et al. 2019). To date, only four *SWEET* genes have been characterized, and they all encode proteins located in plasma membrane (Wang et al. 2019, 2020; Miao et al. 2020).

Based on two different panels, *GmSWEET39/GmSWEET10a* (*Glyma.15g049200*), which located in a selective sweep on chromosome 15, was found to influence the seed oil content. These indicated that *GmSWEET39/GmSWEET10a* may be the domesticated gene (Miao et al. 2020; Wang et al. 2020). It is expressed in the seeds, leaves, pods, and preferentially in the thick-wall parenchyma of the seed coat, where it plays an important role in sucrose translocation to the embryo (Thorne 1981; Henk 1995; Wang et al. 2019; Miao et al. 2020). The natural polymorphisms in the promoter and CDS of *GmSWEET10a* could be used to divide the accessions into at least 12 haplotypes among the two different panels. Based on a median-joining network analysis, these 12 haplotypes were grouped into three major groups: H_I mainly in wild soybeans, H_II mostly in landrace, and H_III primarily in cultivars, respectively (Wang et al. 2019), indicating that *GmSWEET10a* experienced strong selection during domestication and diversification (Miao et al. 2020; Wang et al. 2020). In group H_III, the haplotype H_III_3, which was also named as Hap6, containing the *GmSWEET10a* variants with deletions in the promoter and the CDS and an additional SNP in the CDS, were the potential superior alleles for improving the soybean seed oil content (Miao et al. 2020).

As an ancient polyploid, most soybean genes are present in multiple copies (Schmutz et al. 2010). The paralogous gene for *GmSWEET39/GmSWEET10a* is *GmSWEET24/GmSWEET10b* (*Glyma.08G183500*), which has a highly similar amino acid sequence to *GmSWEET39/ GmSWEET10a* and is similar in function (Miao et al. 2020; Wang et al. 2020); however, *GmSWEET10b* was likely to be selected during diversification (Miao et al. 2020; Wang et al. 2020). Compared with the wild type, the double mutant *sw10a;10b* had reduced glucose, fructose, and sucrose levels in the embryo and an increased sucrose content in the seed coat. *GmSWEET10a/GmSWEET10b* transport sugars to the embryo, triggering embryonic development in the form of cell division and expansion, resulting in larger seeds. The sugar allocation between the seed coat and the embryo influences the carbon resources available for the biosynthesis of acetyl-CoA, which was the precursor for the lipid biosynthesis. This process will expand much energy and limited the protein content (Weber et al. 2005; Wang et al. 2020). According to this theory, *GmSWEET10a/GmSWEET10b* should reduce the protein content because they increase the seed size and oil content of the soybean cultivars, while this phenomenon was only detected in certain genetic backgrounds (Miao et al. 2020; Wang et al. 2020). The possible explanations for this may be that epistasis masks the gene function, or that other *GmSWEET* orthologs compensate for this resource allocation.

## Flowering

Flowering represents the transformation from vegetative growth to the reproductive growth. As a typical short-day (SD) plant, soybean is sensitive to photoperiod, which limited the regions in which this crop could be grown (Cao et al. 2017). Soybean was domesticated from its wild progenitor *G. soja* around the Huang-Huai Valley in central China (Hymowitz and Newell 1981; Han et al. 2016; Wang et al. 2016a). Its dispersal from its region of origin to areas of high latitude meant that the longer daylength delayed soybean flowering and maturity. The suitable growing season in these higher-latitude areas is finite however, requiring farmers to breed early-flowering cultivars suitable for long-day (LD) photoperiods. By contrast, the dispersal of soybean to areas of low latitude meant that the plants flowered early in the shorter photoperiod, resulting in low yields (Lin et al. 2020). This problem also required breeders to develop new cultivars adapted to these novel environmental conditions.

Many studies have explored the mechanisms of flowering in soybean. *E3* and *E4* encode soybean homologs of the *Arabidopsis* photoreceptor *PHYTOCHROME A* (*phyA*) (Liu et al. 2008a; Watanabe et al. 2009). E3 responds to a red light–enriched LD photoperiod (Watanabe et al. 2009). Under the background of *e3* (mutant allele of *E3*), E4 mainly functions in far-red light-enriched LD conditions (Liu et al. 2008a). Both of these proteins receive light signals and function redundantly to regulate downstream genes, including *E1*, encoding a legume-specific transcription factor that plays a core role in the photoperiod network (Xia et al. 2012). Different combinations of the *e3*, *e4*, and *e1* mutant alleles caused partial photoperiod insensitivity under a LD photoperiod (Xu et al. 2013). The florigen gene *FLOWERING LOCUS T* (*FT*) was repressed by E1 (Kong et al. 2010), forming a conserved and key pathway for the flowering time: E3E4–E1–FT (Xia et al. 2012). Subsequent research showed that, in SD environments, *J* (*E6*), a homolog of *Arabidopsis EARLY FLOWERING 3*, could directly bind the promoter of *E1* to repress its expression and induce flowering (Lu et al. 2017). Other genes were also found to participate in the *E3E4–J/E6–E1–FT* pathway, including *E1-Like* (Cober et al. 2010; Zhu et al. 2019), *E2* (*GI*) (Watanabe et al. 2011; Wang et al. 2016c), *CRYPTOCROME* (*CRY*) (Zhang et al. 2008b; Li et al. 2013), *CONSTANS LIKE* (*COL*) (Wu et al. 2014, 2019; Cao et al. 2015a), *miRNA* pathway genes and so on (Cao et al. 2015b; Dong et al. 2021; Li et al. 2021; Zhao et al. 2015). The genes mentioned above were selected by breeders during crop improvement after domestication.

The investigation of the allelic variation of *E2* among 337 accessions, six polymorphic fragments were examined, which resulted in the 47 haplotypes (H1-H47). All 47 haplotypes were found in the wild populations, but only three haplotypes (H1, H2, and H3) were detected in landraces and cultivars. Among the three haplotypes of the domesticated accessions, H1 was most common, and was named *e2* (Wang et al. 2016c). This evidence indicates that *E2* may have been subject to selection, but a lack of molecular evidence remains. Furthermore, in soybean and other plants, there is a continuing debate about whether the flowering trait was selected during domestication (Doebley et al. 2006; Meyer and Purugganan 2013).

Recently, Lu et al. (2020) identified two homologous pseudo-response-regulator (*PRR*) genes, *Tof11* and *Tof12*, which function between *E3/E4* and *E1* to regulate flowering time under LD conditions. *Tof11* and *Tof12* are circadian clock genes that regulate the expression of *E1* through the activity of other members of the circadian clock, the *LATE ELONGATEDHYPOCOTYL* (*LHY*s). Tof11 and Tof12 bind to the promoters of the *LHY*s to suppress their expression, then relieved the transcriptional suppression of E1. Tof11 and Tof12 thus indirectly promote the expression of *E1* to delay flowering, and correspondingly the mutant alleles *tof11* and *tof12* were shown to promote flowering. The analysis of the molecular history of *Tof11* and *Tof12* showed that *Tof12* is located in a selective sweep region, and the most common haplotype, H1 (*tof12-1*), was selected and fixed in most soybean landraces (406/450) and all of the cultivars (532/532) investigated. For *Tof11*, the most common haplotype, H1 (*tof11-1*), was selected and fixed in most cultivars (507/552). Above evidence strongly implied that the selection of *tof11-1* arose in the *tof12-1* genetic background, and thus that *tof12-1* and *tof11-1* were selected during domestication and diversification, respectively. The changes in the allele frequencies of *Tof11* and *Tof12* indicated that *tof12-1* experienced strong artificial selection during domestication to confer mid-early flowering. After domestication, the landraces underwent diversification, during which the early flowering mutant allele *tof11-1* was further fixed to confer very early flowering. The stepwise selection of *tof12-1* and *tof11-1* produced an early phenology and enabled the cultivars to adjust to high latitude. These findings demonstrate that the short crop growth cycle persists from the original domestication phase, and the selection for earlier flowering/maturity can legitimately be viewed as a core domestication trait.

## Perspective

**Fig. 1:**
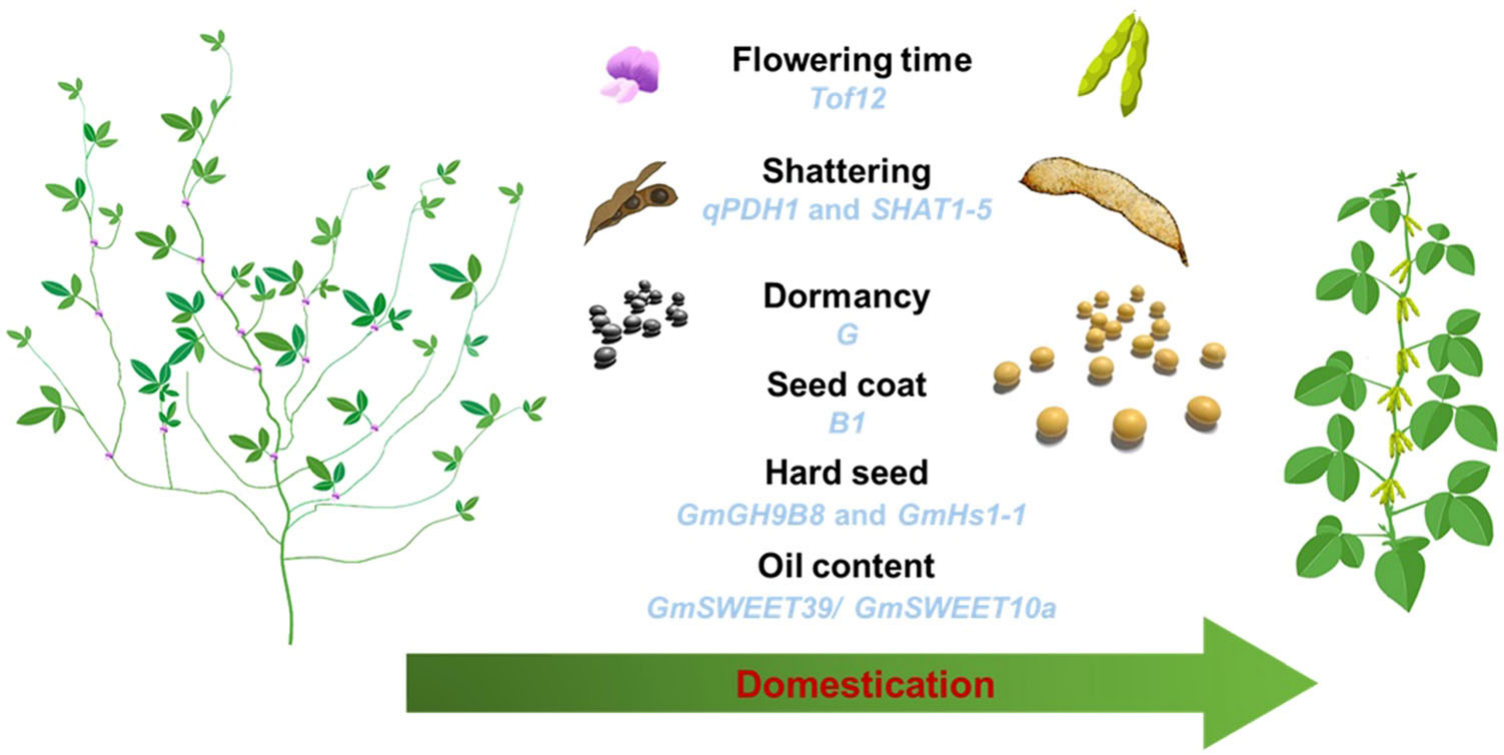
The domestication genes related to domestication traits in soybean. Black text represents the domestication traits. Blue text represents the relevant genes

In this review, we summarized the latest research on key genes associated with domesticated traits in soybean (Fig. 1). Among these genes, only nine genes were positively selected, and at least one causative mutation was fixed within the crop population during domestication and were defined as domesticated genes (Table 1). Early soybean farmers selected the traits useful for themselves, such as seeds without the shattering trait to reduce the yield loss, seeds with a softer coat, dormancy traits to enable sowing and harvesting at the same time, seeds lacking a bloom to avoid human health issues, and plants adapted to a shorter growth period to enable a wider dispersal across the world. In the domestication process, early farmers chose a limited number of elite individuals to use in breeding the next generation, which generated a genetic bottleneck throughout the genome (Doebley et al. 2006). The analysis of the resequencing data showed that approximately half of the genetic diversity and more than 80% of rare alleles were lost during domestication (Burnham et al. 2002; Hyten et al. 2006; Zhou et al. 2015). This raises the question whether the alleles selected for these domesticated genes were truly the best ones.

Soybean is an allotetraploid species, so many of its genes have a duplicated copy in the genome. The above research suggests that usually one of a homologous pair of domesticated genes was selected during domestication, such as *Tof12* or *SWEET10a*, while the other was further selected during improvement, such as *Tof11* and *SWEET10b* (Wang et al. 2019, 2020; Lu et al. 2020; Miao et al. 2020).

Accompanying the selection of the domesticated genes, the genetic diversity of tightly linked genes around them was also lost, potentially losing alleles with beneficial effects on yield or seed quality (Doebley et al. 2006). Scanning for the selective sweeps and digging into these intervals can provide clues not only about new alleles for known genes, but can also help to identify elite genes that may not have been selected in domestication.

Only a limited number of selective sweeps have been performed based on resequence data, and corresponding domesticated traits and genes have not been identified for most of the selective intervals (Zhou et al. 2015; Han et al. 2016). This may attribute to the difficulties of detecting domesticated traits; for example, the twinning habit were only existed in wild soybean and there are no evaluation criteria for this trait, so related statistical data are difficult to obtain. In addition, some domesticated traits are influenced by the environment and have different phenotypes in different periods, which therefore increased the difficulties of performing statistics on the phenotype data. These statistics are therefore just as important as the re-sequence data itself in the detection of the selective sweeps.

Compared with genomics, the use of genetics also has some advantages; for example, RILs can not only be used to detect genetics effects, such as epistatic, additive, and dominant effects, but also to perform fine mapping on the candidate genes. Chromosome segment substitution lines can allow the elimination of noise from the background and the detection of the functions of individual genes, making them another powerful population, especially for the detection of minor effect genes. Current research into domesticated traits and genes is moving towards the use of a combination of genomics and genetics.

The development of whole-genome sequencing enabled the de novo assembly of wild soybean genomes and the resequencing of some soybean accessions, providing more information about the differences between wild and cultivated soybean lines. This provided a pool of data from which we could identify new alleles of putative domesticated genes. In addition, the detection capability of the copy-number variations and presence–absence variations were increased, which further facilitate research into domestication. More in-depth research into domestication will result in the detection of more genes that have undergone parallel selection across different plant families, such as the analyses in rice, tomato (*Solanum lycopersicum*), and *Arabidopsis* indicated that G orthologs were functionally conserved and underwent parallel selection in different families (Wang et al. 2018; Lin et al. 2012). This provided new genetic resources for the further de novo domestication of new crops (Yu et al. 2021). This could finally alleviate the threat of our food supply outstripping demand.

## Data Availability

Data sharing not applicable to this article as no datasets were generated or analyzed during the current study.

## References

[CR1] Bailey MA, Mian MAR, Cater TE, Ashley DA, Boema HR (1997). Pod dehiscence of soybean: identification of quantitative trait loci. J Hered.

[CR2] Baskin JM, Baskin CC (2004). A classification system for seed dormancy. Seed Sci Res.

[CR3] Bewley JD (1997). Seed germination and dormancy. Plant Cell.

[CR4] BirgitKucera MACGL-M (2005). Plant hormone interactions during seed dormancy release and germination. Seed Sci Res.

[CR5] Burnham KD, Francis DM, Dorrance AE, Fioritto RJ, St Martin SK (2002). Genetic diversity patterns among phytophthora resistant soybean plant introductions based on SSR markers. Crop Sci.

[CR6] Cao D, Li Y, Lu S, Wang J, Nan H, Li X, Shi D, Fang C, Zhai H, Yuan X, Anai T, Xia Z, Liu B, Kong F (2015). GmCol1a and GmCol1b function as flowering repressors in soybean under long-day conditions. Plant Cell Physiol.

[CR7] Cao D, Li Y, Wang J, Nan H, Wang Y, Lu S, Jiang Q, Li X, Shi D, Fang C, Yuan X, Zhao X, Li X, Liu B, Kong F (2015). GmmiR156b overexpression delays flowering time in soybean. Plant Mol Biol.

[CR8] Cao D, Takeshima R, Zhao C, Liu B, Jun A, Kong F (2017). Molecular mechanisms of flowering under long days and stem growth habit in soybean. J Exp Bot.

[CR9] Carter TE, Hymowitz T, Nelson RL, Werner D (2004). Biogeography, local adaptation, vavilov, and genetic diversity in soybean. Biological resources and migration.

[CR10] Chen L, Lin IW, Qu X-Q, Sosso D, McFarlane HE, Londono A, Samuels AL, Frommer WB (2015). A cascade of sequentially expressed sucrose transporters in the seed coat and endosperm provides nutrition for the *Arabidopsis* embryo. Plant Cell.

[CR11] Chen L, Qu X, Hou B, Sosso D, Osorio S, Fernie AR, Frommer WB (2012). Sucrose efflux mediated by sweet proteins as a key step for phloem transport. Science.

[CR12] Chen A, Shoemaker RC (1998). Four genes affecting seed traits in soybeans map to linkage group F. J Hered.

[CR13] Christiansen LC, Degan FD, Ulvskov P, Borkhardt B (2002). Examination of the dehiscence zone in soybean pods and isolation of a dehiscence-related endopolygalacturonase gene. Plant Cell Environ.

[CR14] Clemente TE, Cahoon EB (2009). Soybean oil: genetic approaches for modification of functionality and total content. Plant Physiol.

[CR15] Cober ER, Molnar SJ, Charette M, Voldeng HD (2010). A new locus for early maturity in soybean. Crop Sci.

[CR16] Cyrek M, Fedak H, Ciesielski A, Guo Y, Sliwa A, Brzezniak L, Krzyczmonik K, Pietras Z, Kaczanowske S, Liu F, Swiezewski S (2016). Seed dormancy in *Arabidopsis* is controlled by alternative polyadenylation of *DOG1*. Plant Physiol.

[CR17] Dalling JW, Davis AS, Schutte BJ, Arnold AE (2011). Seed survival in soil: Interacting effects of predation, dormancy and the soil microbial community. J Ecol.

[CR18] Doebley JF, Gaut BS, Smith BD (2006). The molecular genetics of crop domestication. Cell.

[CR20] Dong Y, Yang X, Liu J, Wang B, Liu B, Wang Y (2014). Pod shattering resistance associated with domestication is mediated by a *NAC* gene in soybean. Nat Commun.

[CR19] Dong L, Fang C, Cheng Q, Su T, Kou K, Kong L, Zhang C, Li H, Hou Z, Zhang Y, Chen L, Yue L, Wang L, Wang K, Li Y, Gan Z, Yuan X, Weller JL, Lu S, Kong F, Liu B (2021). Genetic basis and adaptation trajectory of soybean from its temperate origin to tropics. Nat Commun.

[CR21] Ferrandiz C, Liljegren SJ, Yanofsky MF (2000). Negative regulation of the *SHATTERPROOF* genes by *FRUITFULL* during Arabidopsis fruit development. Science.

[CR22] Finch-Savage WE, Leubner-Metzger GL (2006). Seed dormancy and the control of germination. New Phytol.

[CR23] Foley ME, Fennimore SA (1998). Genetic basis for seed dormancy. Seed Sci Res.

[CR24] Funatsuki H, Hajika M, Hagihara S, Yamada T, Tanaka Y, Tsuji H, Ishimoto M, Fujino K (2008). Confirmation of the location and the effects of a major QTL controlling pod dehiscence, *qPDH1*, in soybean. Breed Sci.

[CR25] Funatsuki H, Hajika M, Yamada T, Suzuki M, Hagihara S, Tanaka Y, Fujita S, Ishimoto M, Fujino K (2011). Mapping and use of QTLs controlling pod dehiscence in soybean. Breed Sci.

[CR26] Funatsuki H, Suzuki M, Hirose A, Inaba H, Yamada T, Hajika M, Komatsu K, Katayama T, Sayama T, Ishimoto M, Fujino K (2014). Molecular basis of a shattering resistance boosting global dissemination of soybean. Proc Natl Acad Sci USA.

[CR27] Funatsuki H, Ishimoto M, Tsuji H, Kawaguchi K, Hajika M, Fujino K (2006). Simple sequence repeat markers linked to a major QTL controlling pod shattering in soybean. Plant Breed.

[CR28] Funatsuki H, Kawaguchi K, Matsuba S, Sato Y, Ishimoto M (2005). Mapping of QTL associated with chilling tolerance during reproductive growth in soybean. Theor Appl Genet.

[CR29] Gao M, Zhu H (2013). Fine mapping of a major quantitative trait locus that regulates pod shattering in soybean. Mol Breed.

[CR30] Gijzen M, Miller SS, Kuflu K, Buzzell RI, Miki BLA (1999). Hydrophobic protein synthesized in the pod endocarp adheres to the seed surface. Plant Physiol.

[CR31] Graham PH, Vance CP (2003). Legumes: Importance and constraints to greater use. Plant Physiol.

[CR32] Hammer K (1984). Das domestikationssyndrom. Die. Kulturpflanze.

[CR33] Han Y, Zhao X, Liu D, Li Y, Lightfoot DA, Yang Z, Zhao L, Zhou G, Wang Z, Huang L, Zhang Z, Qiu L, Zheng H, Li W (2016). Domestication footprints anchor genomic regions of agronomic importance in soybeans. New Phytol.

[CR34] Henk HW (1995). A critical update on seed dormancy I Primary dormancy. Seed Sci Res.

[CR35] Henrissat B (1991). A classification of glycosyl hydrolases based on amino acid sequence similarities. Biochem J.

[CR36] Hilhorst HWM (1995). A critical update on seed dormancy I Primary dormancy. Seed Sci Res.

[CR37] Hosmani PS, Kamiya T, Danku J, Naseer S, Geldner N, Guerinot ML, Salt DE (2013). Dirigent domain-containing protein is part of the machinery required for formation of the lignin-based Casparian strip in the root. Proc Natl Acad Sci USA.

[CR38] Hymowitz T, Newell CA (1981). Taxonomy of the genus glycine, domestication and uses of soybeans. Econ Bot.

[CR39] Hyten DL, Song Q, Zhu Y, Choi I-Y, Nelson RL, Costa JM, Specht JE, Shoemaker RC, Cregan PB (2006). Impacts of genetic bottlenecks on soybean genome diversity. Proc Natl Acad Sci USA.

[CR40] Jang S, Sato M, Sato K, Jitsuyama Y, Fujino K, Mori H, Takahashi R, Benitez ER, Liu B, Yamada T, Abe J (2015). A single-nucleotide polymorphism in an endo-1,4-β-glucanase gene controls seed coat permeability in soybean. PLoS ONE.

[CR41] Kang S, Kim HK, Baek IY, Chung MG, Young W, Shin DC, Lee S-H (2005). Genetic analysis of pod dehiscence in soybean. Korean J Crop Sci.

[CR42] Keim P, Diers BW, Shoemaker RC (1990). Genetic analysis of soybean hard seededness with molecular markers. Theoret Appl Genet.

[CR43] Kim KW, Moinuddin SGA, Atwell KM, Costa MA, Davin LB, Lewis NG (2012). Opposite stereoselectivities of dirigent proteins in *Arabidopsis* and Schizandra species. J Biol Chem.

[CR44] Kim MY, Van K, Kang YJ, Kim KH, Lee SH (2011). Tracing soybean domestication history: from nucleotide to genome. Breed Sci.

[CR45] Kong F, Liu B, Xia Z, Sato S, Kim BM, Watanabe S, Yamada T, Tabata S, Kanazawa A, Harada K, Abe J (2010). Two coordinately regulated homologs of *FLOWERING LOCUS T* are involved in the control of photoperiodic flowering in soybean. Plant Physiol.

[CR46] Koornneef M, Bentsink L, Hilhorst H (2002). Seed dormancy and germination. Curr Opin Plant Biol.

[CR47] Kucera B, Cohn M, Leubner-Metzger G (2005). Plant hormone interactions during seed dormancy release and germination. Seed Sci Res.

[CR48] Lee JS, Kim KR, Ha B-K, Kang S (2017). Identification of SNPs tightly linked to the QTL for pod shattering in soybean. Mol Breed.

[CR49] Lenser T, Theißen G (2013). Molecular mechanisms involved in convergent crop domestication. Trends Plant Sci.

[CR51] Li B, Foley ME (1997). Genetic and molecular control of seed dormancy. Trends Plant Sci.

[CR52] Li Y, Zhao S, Ma J, Li D, Yan L, Li J, Qi X, Guo X, Zhang L, He W, Chang R, Liang Q, Guo Y, Ye C, Wang X, Tao Y, Guan R, Wang J, Liu Y, Jin L, Zhang X, Liu Z, Zhang L, Chen J, Wang K, Nielsen R, Li R, Chen P, Li W, Reif JC, Purugganan M, Wang J, Zhang M, Wang J, Qiu L (2013). Molecular footprints of domestication and improvement in soybean revealed by whole genome re-sequencing. BMC Genom.

[CR50] Li X, Fang C, Yang Y, Lv T, Su T, Chen L, Nan H, Li S, Zhao X, Lu S, Dong L, Cheng Q, Tang Y, Xu M, Abe J, Hou X, Weller JL, Kong F, Liu B (2021). Overcoming the genetic compensation response of soybean florigens to improve adaptation and yield at low latitudes. Curr Biol.

[CR53] Libertini E, Li Y, McQueen-Mason SJ (2004). Phylogenetic analysis of the plant endo-beta-1,4-glucanase gene family. J Mol Evo.

[CR54] Liljegren SJ, Ditta GS, Eshed HY, Savidge B, Bowman JL, Yanofsky MF (2000). *SHATTERPROOF* MADS-box genes control seed dispersal in *Arabidopsis*. Nature.

[CR55] Lin Z, Li X, Shannon LM, Yeh CT, Wang M, Bai G, Peng Z, Li J, Trick HN, Clemente TE, Doebley J, Schnable PS, Tuinstra MR, Tesso TT, White F, Yu J (2012). Parallel domestication of the *Shattering1* genes in cereals. Nat Genet.

[CR56] Lin IW, Sosso D, Chen L, Gase K, Kim SG, Kessler D, Klinkenberg PM, Gorder MK, Hou B, Qu X, Carter CJ, Baldwin IT, Frommer WB (2014). Nectar secretion requires sucrose phosphate synthases and the sugar transporter *SWEET9*. Nature.

[CR57] Lin X, Liu B, Weller JL, Abe J, Kong F (2020). Molecular mechanisms for the photoperiodic regulation of flowering in soybean. J Integr Plant Biol.

[CR58] Liu B, Fujita T, Yan ZH, Sakamoto S, Xu D, Abe J (2007). QTL mapping of domestication-related traits in soybean (*glycine max*). Ann Bot.

[CR59] Liu B, Kanazawa A, Matsumura H, Takahashi R, Harada K, Abe J (2008). Genetic redundancy in soybean photoresponses associated with duplication of the phytochrome a gene. Genetics.

[CR60] Liu J, Stipanovic RD, Bell AA, Puckhaber LS, Magill CW (2008). Stereoselective coupling of hemigossypol to form (+)-gossypol in moco-cotton is mediated by a dirigent protein. Phytochemistry.

[CR61] Lu S, Dong L, Fang C, Liu S, Kong L, Cheng Q, Chen L, Su T, Nan H, Zhang D, Zhang L, Wang Z, Yang Y, Yu D, Liu X, Yang Q, Lin X, Tang Y, Zhao X, Yang X, Tian C, Xie Q, Li X, Yuan X, Tian Z, Liu B, Weller JL, Kong F (2020). Stepwise selection on homeologous *PRR* genes controlling flowering and maturity during soybean domestication. Nat Genet.

[CR62] Lu S, Zhao X, Hu Y, Liu S, Nan H, Li X, Fang C, Cao D, Shi X, Kong L, Su T, Zhang F, Li S, Wang Z, Yuan X, Cober ER, Weller JL, Liu B, Hou X, Tian Z, Kong F (2017). Natural variation at the soybean *J* locus improves adaptation to the tropics and enhances yield. Nat Genet.

[CR63] Ma F, Cholewa E, Mohamed T, Peterson CA, Gijzen M (2004). Cracks in the palisade cuticle of soybean seed coats correlate with their permeability to water. Ann Bot.

[CR64] McKibbin MHSKR (1999). Molecular and genetic mechanisms regulating the transition from embryo development to germination. Trends Plant Sci.

[CR65] Meyer RS, Purugganan MD (2013). Evolution of crop species: genetics of domestication and diversification. Nat Rev Genet.

[CR66] Miao L, Yang S, Zhang K, He J, Wu C, Ren Y, Gai J, Li Y (2020). Natural variation and selection in *GmSWEET39* affect soybean seed oil content. New Phytol.

[CR67] Miranda C, Culp C, Skrabisova M, Joshi T, Belzile F, Grant DM, Bilyeu K (2019). Molecular tools for detecting *Pdh1* can improve soybean breeding efficiency by reducing yield losses due to pod shatter. Mol Breed.

[CR68] Mitsuda N, Seki M, Shinozaki K, Ohme-Takagi M (2005). The Nac transcription factors NST1 and NST2 of *Arabidopsis* regulate secondary wall thickenings and are required for anther dehiscence. Plant Cell.

[CR69] Molhoj M, Pagant SR, Hofte H (2002). Towards understanding the role of membrane-bound endo-beta-1,4-glucanases in cellulose biosynthesis. Plant Cell Physiol.

[CR70] Nakabayashi K, Bartsch M, Ding J, Soppe WJJ (2015). Seed dormancy in *Arabidopsis* requires self-binding ability of DOG1 protein and the presence of multiple isoforms generated by alternative splicing. PLoS Genet.

[CR71] Née G, Kramer K, Nakabayashi K, Yuan B, Xiang Y, Miatton E, Finkemeier I, Soppe WJJ (2017). DELAY OF GERMINATION1 requires PP2C phosphatases of the ABA signalling pathway to control seed dormancy. Nat Commun.

[CR72] Newell CA, Hymowitz T (1978). Seed coat variation in *Glycine* Willd. subgenus *Glycine* (Leguminosae) by SEM. Brittonia.

[CR73] Nishizawa K, Kita Y, Kitayama M, Ishimoto M (2006). A red fluorescent protein, DsRed2, as a visual reporter for transient expression and stable transformation in soybean. Plant Cell Rep.

[CR74] Ogawa M, Kay P, Wilson S, Swain SM (2009). ARABIDOPSIS DEHISCENCE ZONE POLYGALACTURONASE1 (ADPG1), ADPG2, and QUARTET2 are polygalacturonases required for cell separation during reproductive development in *Arabidopsis*. Plant Cell.

[CR75] Olsen OA (2001). Endosperm development: cellularization and cell fate specification. Annu Rev Plant Biol.

[CR76] Olsen KM, Wendel JF (2013). A bountiful harvest: genomic insights into crop domestication phenotypes. Annu Rev Plant Biol.

[CR77] Pallais N (1995). Storage factors control germination and seedling establishment of freshly harvested true potato seed. American Potato J.

[CR78] Patrick JW, Offler CE (2001). Compartmentation of transport and transfer events in developing seeds. J Exp Bot.

[CR79] Paulsen TR, Colville L, Kranner I, Daws MI, Hogstedt G, Vandvik V, Thompson K (2013). Physical dormancy in seeds: a game of hide and seek?. New Phyto.

[CR80] Pickel B, Constantin M-A, Pfannstiel J, Conrad J, Beifuss U, Schaller A (2010). An enantiocomplementary dirigent protein for the enantioselective laccase-catalyzed oxidative coupling of phenols. Angew Chem Int Ed.

[CR81] Porter SS (2013). Adaptive divergence in seed color camouflage in contrasting soil environments. New Phytol.

[CR82] Rajani S, Sundaresan V (2001). The *Arabidopsis* myc/bHLH gene ALCATRAZ enables cell separation in fruit dehiscence. Curr Biol.

[CR83] Ruan Y-L, Patrick JW, Bouzayen M, Osorio S, Fernie AR (2012). Molecular regulation of seed and fruit set. Trends Plant Sci.

[CR84] Sakamoto SI, Abe J, Kanazawa A, Shimamoto Y (2004). Marker-assisted analysis for soybean hard seededness with isozyme and simple sequence repeat loci. Breed Sci.

[CR85] Schmutz J, Cannon SB, Schlueter J, Ma J, Mitros T, Nelson W, Hyten DL, Song Q, Thelen JJ, Cheng J, Xu D, Hellsten U, May GD, Yu Y, Sakurai T, Umezawa T, Bhattacharyya MK, Sandhu D, Valliyodan B, Lindquist E, Peto M, Grant D, Shu S, Goodstein D, Barry K, Futrell-Griggs M, Abernathy B, Du J, Tian Z, Zhu L, Gill N, Joshi T, Libault M, Sethuraman A, Zhang X, Shinozaki K, Nguyen HT, Wing RA, Cregan P, Specht J, Grimwood J, Rokhsar D, Stacey G, Shoemaker RC, Jackson SA (2010). Genome sequence of the palaeopolyploid soybean. Nature.

[CR86] Shao S, Meyer CJ, Ma F, Peterson CA, Bernards MA (2007). The outermost cuticle of soybean seeds: chemical composition and function during imbibition. J Exp Bot.

[CR87] Simons KJ, Fellers JP, Trick HN, Zhang ZC, Tai YS, Gill BS, Faris JD (2006). Molecular characterization of the major wheat domestication gene *Q*. Genetics.

[CR88] Sorefan K, Girin T, Liljegren SJ, Ljung K, Robles P, Galván-Ampudia CS, Offringa R, Friml J, Yanofsky MF, Østergaard L (2009). A regulated auxin minimum is required for seed dispersal in *Arabidopsis*. Nature.

[CR89] Spence J, Vercher Y, Gates P, Harris N (1996). ‘Pod shatter’ in *Arabidopsis thaliana*, *Brassica napus* and *B. juncea*. J Microsc.

[CR90] Sugimoto K, Takeuchi Y, Ebana K, Miyao A, Hirochika H, Hara N, Ishiyama K, Kobayashi M, Ban Y, Hattori T, Yano M (2010). Molecular cloning of *Sdr4*, a regulator involved in seed dormancy and domestication of rice. Proc Natl Acad Sci USA.

[CR91] Sun M, Huang X, Yang J, Guan Y, Yang Z (2013). *Arabidopsis* RPG1 is important for primexine deposition and functions redundantly with RPG2 for plant fertility at the late reproductive stage. Plant Reprod.

[CR92] Sun L, Miao Z, Cai C, Zhang D, Zhao M, Wu Y, Zhang X, Swarm SA, Zhou L, Zhang ZJ, Nelson RL, Ma J (2015). *GmHs1-1*, encoding a calcineurin-like protein, controls hard-seededness in soybean. Nat Genet.

[CR93] Sun X, Shantharaj D, Kang X, Ni M (2010). Transcriptional and hormonal signaling control of *Arabidopsis* seed development. Curr Opin Plant Biol.

[CR94] Suzuki M, Fujino K, Nakamoto Y, Ishimoto M, Funatsuki H (2010). Fine mapping and development of DNA markers for the *qPDH1* locus associated with pod dehiscence in soybean. Mol Breed.

[CR95] Thorne JH (1981). Morphology and ultrastructure of maternal seed tissues of soybean in relation to the import of photosynthate. Plant Physiol.

[CR96] Tischner T, Allphin L, Chase K, Orf JH, Lark KG (2003). Genetics of seed abortion and reproductive traits in soybean. Crop Sci.

[CR97] Tiwari ST, Bhatia VS (1995). Characters of pod anatomy associated with resistance to pod-shattering in soybean. Ann Bot.

[CR98] Urbanowicz BR, Bennett AB, del Campillo E, Catala C, Hayashi T, Henrissat B, Hoefte H, McQueen-Mason SJ, Patterson SE, Shoseyov O, Teeri TT, Rose JKC (2007). Structural organization and a standardized nomenclature for plant endo-1,4-B-glucanases (cellulases) of glycosyl hydrolase family 9. Plant Physiol.

[CR101] Wang K, Li X, Li F (2008). Phenotypic diversity of the big seed type subcollection of wild soybean (*Glycine soja* Sieb. et Zucc.) in China. Genet Resour Crop Evol.

[CR99] Wang J, Chu S, Zhang H, Zhu Y, Cheng H, Yu D (2016). Development and application of a novel genome-wide snp array reveals domestication history in soybean. Sci Rep.

[CR102] Wang W, Li X, Chen S, Song S, Gai J, Zhao T (2016). Using presence/absence variation markers to identify the QTL/allele system that confers the small seed trait in wild soybean (*Glycine soja* Sieb. & Zucc.). Euphytica.

[CR100] Wang Y, Gu Y, Gao H, Qiu L, Chang R, Chen S, He C (2016). Molecular and geographic evolutionary support for the essential role of *GIGANTEAa* in soybean domestication of flowering time. BMC Evol Biol.

[CR103] Wang M, Li W, Fang C, Xu F, Liu Y, Wang Z, Yang R, Zhang M, Liu S, Lu S, Lin T, Tang J, Wang Y, Wang H, Lin H, Zhu B, Chen M, Kong F, Liu B, Zeng D, Jackson SA, Chu C, Tian Z (2018). Parallel selection on a dormancy gene during domestication of crops from multiple families. Nat Genet.

[CR104] Wang S, Liu S, Wang J, Yokosho K, Zhou B, Yu Y, Liu Z, Frommer WB, Ma JF, Chen L, Guan Y, Shou H, Tian Z (2020). Simultaneous changes in seed size, oil content and protein content driven by selection of *SWEET* homologues during soybean domestication. Natl Sci Rev.

[CR105] Wang L, Ruan Y (2012). New insights into roles of cell wall invertase in early seed development revealed by comprehensive spatial and temporal expression patterns of *GhCWIN1* in cotton. Plant Physiol.

[CR106] Wang S, Yokosho K, Guo R, Whelan J, Ruan Y, Ma JF, Shou H (2019). The soybean sugar transporter *GmSWEET15* mediates sucrose export from endosperm to early embryo. Plant Physiol.

[CR107] Watanabe S, Hideshima R, Xia Z, Tsubokura Y, Sato S, Nakamoto Y, Yamanaka N, Takahashi R, Ishimoto M, Anai T, Tabata S, Harada K (2009). Map-based cloning of the gene associated with the soybean maturity locus *E3*. Genetics.

[CR108] Watanabe S, Tajuddin T, Yamanaka N, Hayashi M, Harada K (2004). Analysis of QTLs for reproductive development and seed quality traits in soybean using recombinant inbred lines. Breed Sci.

[CR109] Watanabe S, Xia Z, Hideshima R, Tsubokura Y, Sato S, Yamanaka N, Takahashi R, Anai T, Tabata S, Kitamura K, Harada K (2011). A map-based cloning strategy employing a residual heterozygous line reveals that the *GIGANTEA* gene is involved in soybean maturity and flowering. Genetics.

[CR110] Weber H, Borisjuk L, Wobus U (2005). Molecular physiology of legume seed development. Annu Rev Plant Biol.

[CR112] Wu F, Kang X, Wang M, Haider W, Price WB, Hajek B, Hanzawa Y (2019). Transcriptome-enabled network inference revealed the *GmCOL1* feed-forward loop and its roles in photoperiodic flowering of soybean. Front Plant Sci.

[CR113] Wu F, Price BW, Haider W, Seufferheld G, Nelson R, Hanzawa Y (2014). Functional and evolutionary characterization of the *CONSTANS* gene family in short-day photoperiodic flowering in soybean. PLoS ONE.

[CR114] Xia Z, Watanabe S, Yamada T, Tsubokura Y, Nakashima H, Zhai H, Anai T, Sato S, Yamazaki T, Lu S, Wu H, Tabata S, Harada K (2012). Positional cloning and characterization reveal the molecular basis for soybean maturity locus *E1* that regulates photoperiodic flowering. Proc Natl Acad Sci USA.

[CR115] Xu M, Xu Z, Liu B, Kong F, Tsubokura Y, Watanabe S, Xia Z, Harada K, Kanazawa A, Yamada T, Abe J (2013). Genetic variation in four maturity genes affects photoperiod insensitivity and phya-regulated post-flowering responses of soybean. BMC Plant Biol.

[CR116] Xuan YH, Hu YB, Chen L-Q, Sosso D, Ducat DC, Hou B-H, Frommer WB (2013). Functional role of oligomerization for bacterial and plant SWEET sugar transporter family. Proc Natl Acad Sci USA.

[CR117] Yang Y, Zheng C, Chandrasekaran U, Yu L, Liu C, Pu T, Wang X, Du J, Liu J, Yang F, Yong T, Yang W, Liu W, Shu K (2020). Identification and bioinformatic analysis of the *GmDOG1-Like* family in soybean and investigation of their expression in response to gibberellic acid and abscisic acid. Plants.

[CR118] Yu H, Lin T, Meng X, Du H, Zhang J, Liu G, Chen M, Jing Y, Kou L, Li X, Gao Q, Liang Y, Liu X, Fan Z, Liang Y, Cheng Z, Chen M, Tian Z, Wang Y, Chu C, Zuo J, Wan J, Qian Q, Han B, Zuccolo A, Wing RA, Gao C, Liang C, Li J (2021). A route to de novo domestication of wild allotetraploid rice. Cell.

[CR119] Yuan M, Wang S (2013). Rice MtN3/Saliva/SWEET family genes and their homologs in cellular organisms. Mol Plant.

[CR120] Zhang B, Chen P, Chen CY, Wang D, Shi A, Hou A, Ishibashi T (2008). Quantitative trait loci mapping of seed hardiness in soybean. Crop Sci.

[CR121] Zhang Q, Li H, Li R, Hu R, Fan C, Chen F, Wang Z, Liu X, Fu Y, Lin C (2008). Association of the circadian rhythmic expression of *GmCRY1a* with a latitudinal cline in photoperiodic flowering of soybean. Proc Natl Acad Sci USA.

[CR122] Zhang D, Sun L, Li S, Wang W, Ding Y, Swarm SA, Li L, Wang X, Tang X, Zhang Z, Tian Z, Brown PJ, Cai C, Nelson RL, Ma J (2018). Elevation of soybean seed oil content through selection for seed coat shininess. Nat Plants.

[CR123] Zhao X, Cao D, Huang Z, Wang J, Lu S, Xu Y, Liu B, Kong F, Yuan X (2015). Dual functions of GmTOE4a in the regulation of photoperiod-mediated flowering and plant morphology in soybean. Plant Mol Biol.

[CR124] Zhou Z, Jiang Y, Wang Z, Gou Z, Lyu J, Li W, Yu Y, Shu L, Zhao Y, Ma Y, Fang C, Shen Y, Liu T, Li C, Li Q, Wu M, Wang M, Wu Y, Dong Y, Wan W, Wang X, Ding Z, Gao Y, Xiang H, Zhu B, Lee SH, Wang W, Tian Z (2015). Resequencing 302 wild and cultivated accessions identifies genes related to domestication and improvement in soybean. Nat Biotechnol.

[CR125] Zhou Y, Lu D, Li C, Luo J, Zhu B-F, Zhu J, Shangguan Y, Wang Z, Sang T, Zhou B, Han B (2012). Genetic control of seed shattering in rice by the APETALA2 transcription factor *SHATTERING ABORTION 1*. Plant Cell.

[CR126] Zhu J, Takeshima R, Harigai K, Xu M, Kong F, Liu B, Kanazawa A, Yamada T, Abe J (2019). Loss of function of the *E1-like-b* gene associates with early flowering under long-day conditions in soybean. Front Plant Sci.

