# Correction: Current overview on the genetic basis of key genes involved in soybean domestication

**DOI:** 10.1007/s42994-024-00161-9

**Published:** 2024-04-30

**Authors:** Sijia Lu, Chao Fang, Jun Abe, Fanjiang Kong, Baohui Liu

**Affiliations:** 1 Innovative Center of Molecular Genetics and Evolution, School of Life Sciences, Guangzhou University, Guangzhou, 510006 China; 2 Guangzhou Key Laboratory of Crop Gene Editing, Guangzhou University, Guangzhou, 510006 China; 3 Research Faculty of Agriculture, Hokkaido University, Sapporo, Hokkaido 060-0808 Japan

**Correction: aBIOTECH (2022) 3:126–139** 10.1007/s42994-022-00074-5

The article “Current overview on the genetic basis of key genes involved in soybean domestication”, written by Sijia Lu, Chao Fang, Jun Abe, Fanjiang Kong and Baohui Liu, was originally published Online First without Open Access. After publication in volume 3, issue 2, pages 126–139 the authors decided to opt for Open Choice and to make the article an Open Access publication. Therefore, the copyright of the article has been changed to © The Authors 2024 and the article is forthwith distributed under the terms of the Creative Commons Attribution 4.0 International License, which permits use, sharing, adaptation, distribution and reproduction in any medium or format, as long as you give appropriate credit to the original author(s) and the source, provide a link to the Creative Commons licence, and indicate if changes were made.

The images or other third party material in this article are included in the article’s Creative Commons licence, unless indicated otherwise in a credit line to the material. If material is not included in the article’s Creative Commons licence and your intended use is not permitted by statutory regulation or exceeds the permitted use, you will need to obtain permission directly from the copyright holder.

To view a copy of this licence, visit http://creativecommons.org/licenses/by/4.0/.

